# Disciplinary trends in the use of the Delphi method: A bibliometric analysis

**DOI:** 10.1371/journal.pone.0289009

**Published:** 2023-08-15

**Authors:** Dmitry Khodyakov, Sean Grant, Jack Kroger, Catria Gadwah-Meaden, Aneesa Motala, Jody Larkin

**Affiliations:** 1 RAND Corporation, Santa Monica, California, United States of America; 2 HEDCO Institute for Evidence-Based Educational Practice, College of Education, University of Oregon, Eugene, Oregon, United States of America; 3 Pardee RAND Graduate School, Santa Monica, California, United States of America; 4 RAND Corporation, Pittsburgh, Pennsylvania, United States of America; Max Planck Institute for Solid State Research, GERMANY

## Abstract

The Delphi method is an iterative, anonymous, group-based process for eliciting and aggregating opinion on a topic to explore the existence of consensus among experts. The year 2023 marks the 60th anniversary of the first peer-reviewed journal article on the Delphi method. Originally developed for operations research, this method is now applied extensively by researchers representing diverse scientific fields. We used a bibliometric analysis to describe general trends in the expansion of its use across disciplines over time. We conducted a systematic literature search for all English-language, peer-reviewed journal articles on the Delphi method through its first 60 years. We found 19,831 articles: 96.8% (n = 19,204) on the actual use of the Delphi method in an empirical study and 3.2% (n = 627) describing, examining, or providing some guidance on how to use the Delphi method. Almost half (49.9%) of all articles were published in the 2010s and an additional third (32.5%) in the first few years of the 2020s. Nearly two-thirds (65%, n = 12,883) of all published articles have appeared in medical journals, compared to 15% in science and technology (n = 3,053) or social science (n = 3,016) journals. We conclude that the expanded use of the Delphi method has been driven largely by the medical field, though social scientists and technologists continue to be at the forefront of methodological work on the Delphi method. Therefore, we call for greater transdisciplinary collaboration on methodological guidance and standards for the Delphi method.

## Introduction

The Delphi method is an iterative, anonymous, group-based process for eliciting and aggregating opinion on a topic with a goal of exploring the existence of consensus among experts [1]. It involves a series of questionnaires that panelists answer based on their expertise and experience [2]. Questionnaire rounds are interspersed with controlled feedback on the results of the previous round that are shared with all panelists to encourage each to review how their answers compare to the rest of the panel [3]. Panelists then have the opportunity to revise their responses and provide rationales for their new ratings in future rounds [4]. The process continues until stability or consensus is reached or a pre-determined number of rounds is completed [5]. Expert consensus achieved through the Delphi process is considered to be more reliable than the opinion of a single expert because it maximizes the benefits of engaging a group of knowledgeable individuals while mitigating the negative effects of dominant personalities and groupthink [6]. Thus, expert consensus is often treated as “evidence” that, in combination with other data sources, can help make more accurate predictions of the future and inform decision-making process [7].

The year 2023 marks the 60^th^ anniversary of the first peer-reviewed journal article on the Delphi method: “An experimental application of the Delphi method to the use of experts” [8]. Published in *Management Science*, this seminal article introduced the public to a new method for eliciting expert opinion that was created as part of an experiment called “Project Delphi.” Conceived in 1948 and originally called the “Prediction Study,” the goal of “Project Delphi” was to help the United States Air Force formulate more reliable forecasts of future political developments—namely, the effect of technology on warfare. The project team at the RAND Corporation developed the Delphi method to obtain a reliable expert consensus as a substitute for empirical evidence when it did not exist [9]. “Project Delphi” marked the development of a new epistemological approach to explanation and prediction in “inexact sciences”—such as engineering, medicine, and the social sciences—that relies on the systematic examination of expert judgement rather than forecasting methodologies fully based on “the record of past statistics in analogous instances” [7].

Originally developed for operations research in the military, the Delphi method is now applied extensively by researchers representing diverse scientific fields. This article uses a bibliometric analysis to describe general trends in the expansion of its use across disciplines over time [10]. We answer three research questions:

- How frequently has the Delphi method been used?
- What disciplines have used it the most?
- How has its use by different disciplines changed over time?

Our work is the most comprehensive bibliometric analysis of the Delphi method to date that not only covers its complete 60-year history, but also moves beyond a somewhat narrow focus on the use of the Delphi in forecasting that characterized previous bibliographic work on this method [11, 12].

## Materials and methods

To describe how the use of the Delphi method evolved over time, we conducted a bibliometric analysis, which is useful for summarizing large quantities of bibliometric data to identify “the intellectual structure and emerging trends of a research topic or field” [10]. We focused specifically on the total number of publications as the main metric of interest.

We conducted a systematic literature search to identify peer-reviewed journal articles on the Delphi method published in English (see the S1 Appendix for the full search strategy). Because we were interested in all disciplines (rather than those focused on forecasting), we expanded the number of databases and search dates used in previous bibliographic analyses of the Delphi method [11, 12]. We used the term “Delphi” in all citation fields to search 10 databases (ACM Guide to Computing Literature, AMED, Business Source Complete, CINAHL, ERIC, PsycInfo, PubMed, RAND Library Catalog, Scopus, Web of Science) from January 1, 1950 through May 2022.

We removed duplicate citations, reports, proceedings papers and conference abstracts. We identified duplicates using the “find duplicates” query in EndNote and via manual screening. We also excluded citations that were about a town, a company, and a detector called Delphi. After uploading search results to the literature review software DistillerSR (www.evidencepartners.com), we screened titles and abstracts of English-language journal articles that either reported on the actual use of the Delphi method in an empirical study (i.e., “application” articles) or described, examined, or provided guidance on how the Delphi method should be used (i.e., “methodological” articles), which were our inclusion criteria. Cases that were ambiguous or unclear during the initial screening were flagged for review by a second team member.

In contrast to bespoke and somewhat subjective classification systems used in previous bibliographic analyses of the Delphi method [11, 12], we used the main classes of the Library of Congress (LC) Subject Classification (https://www.loc.gov/catdir/cpso/lcco/) to group the journals that published Delphi articles into scientific disciplines (e.g., medicine, science, technology, social science). Using the FirstSearch interface, we searched WorldCat to identify journal titles in the Library of Congress Catalog; if a journal title had no record, we broadened the search to all library catalogs in WorldCat and chose a record that contained an LC classification for that journal title. If a journal was not listed in WorldCat, we manually coded it using the LC classification system. If a journal had more than one classification, we used the first one listed. We combined some disciplines with low numbers of Delphi publications for ease of presentation, focusing our analyses on four categories of disciplines: medicine, social science, science and technology, and other (e.g., education, military science, and arts). For medical journals only, we also identified subclasses (e.g., internal medicine, public aspects of medicine, surgery) to better describe Delphi use in the medical field.

To further describe the growing use of the Delphi method in the medical field, we analyzed the decennial growth trends in the number of published Delphi articles, relative to the growth rates in the number of all English language articles. To do so, we looked at PubMed, the medical database with the highest number of Delphi publications. We focused our analysis on the publication period between 1970 and 2019 to cover five complete decades. For this analysis, we did not include other databases because many articles are listed in more than one database.

We used descriptive statistics to summarize trends by decade and discipline.

## Results

**Fig 1:**
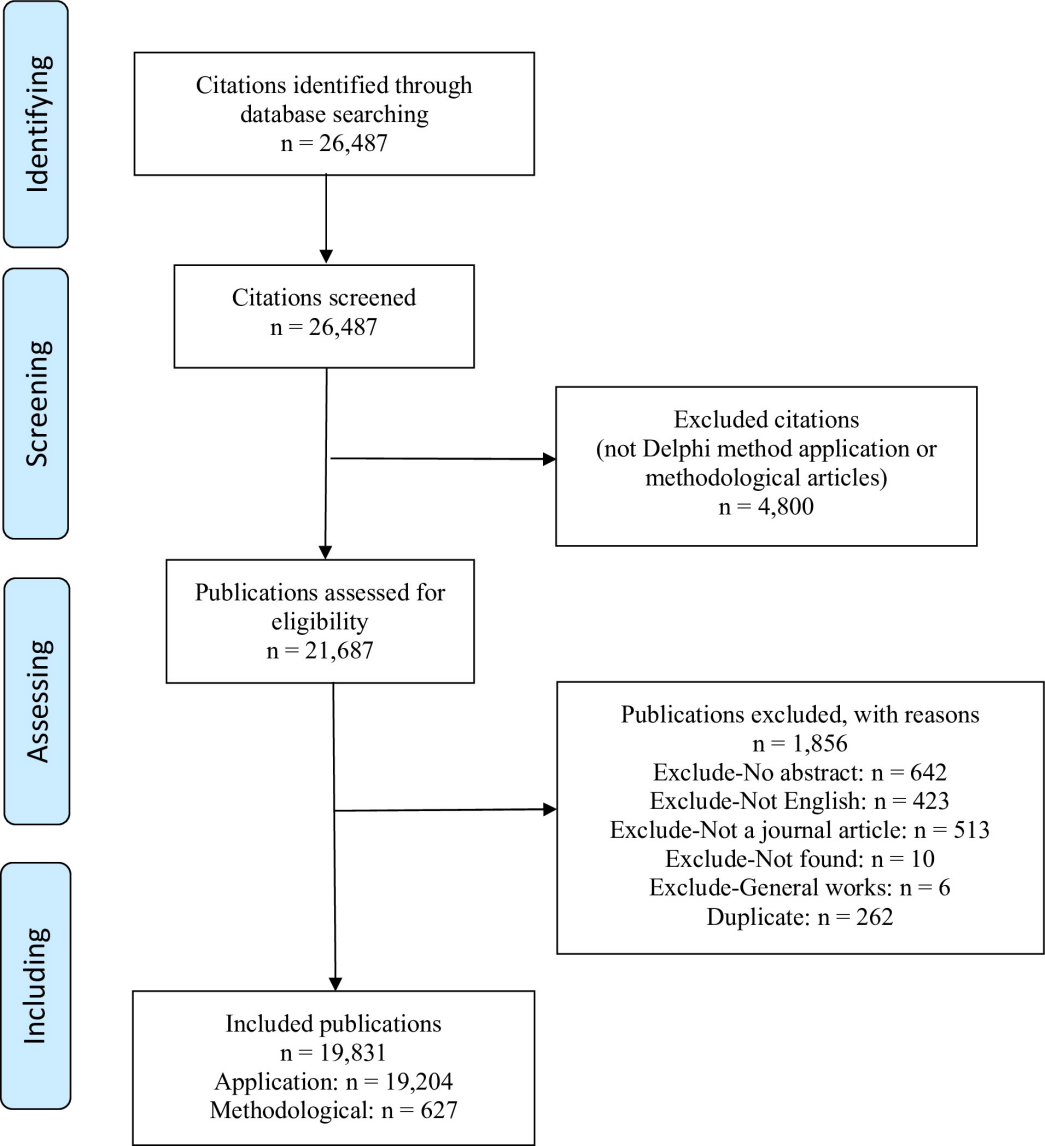
Flow diagram of identifying, screening, assessing, and including studies. This figure shows the number of articles identified, screened, assessed, and included as part of the literature search process.

Our search identified 26,487 citations (Fig 1). After excluding duplicates and articles that did not meet our inclusion criteria, we retained 19,831 unique articles: 96.8% (n = 19,204) were application articles reporting on the actual use of the Delphi method in an empirical study and 3.2% (n = 627) were methodological articles that described, examined, or provided some guidance on how the Delphi method should be used.

### How frequently has the Delphi method been used?

**Fig 2:**
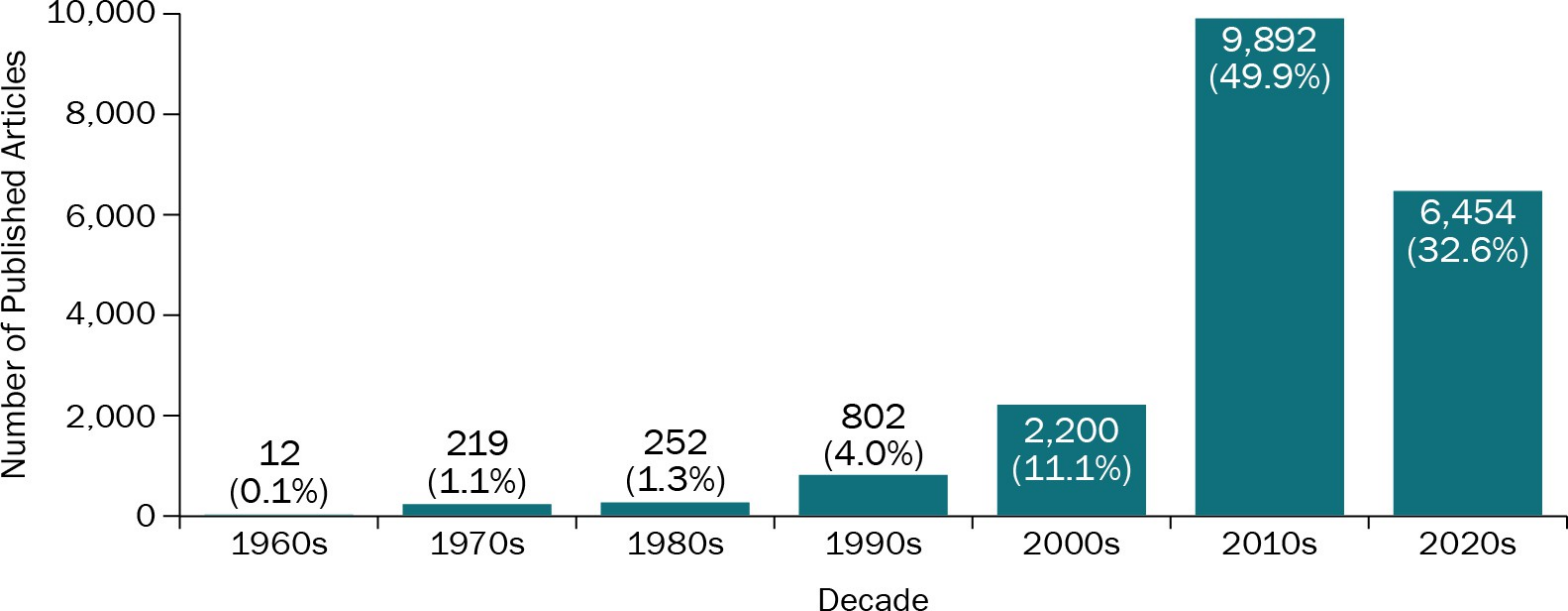
Number of published Delphi articles by decade. This figure shows the number and percent of Delphi articles published in each decade, starting from 1960.

While the first article that mentioned the Delphi method was published in 1963, only 12 Delphi publications appeared in the 1960s. This number increased to 219 in the 1970s and to 252 in the 1980s (1% of all published Delphi articles in each decade). However, there was a steady increase starting in the 1990s: the number of Delphi articles increased to 802 (4.0%) in the last decade of the 20^th^ century and then to 2,200 (11.1%) in the first decade of the 21^st^ century. The number of Delphi publications increased approximately five-fold in the 2010s (n = 9,892), with roughly half (49.9%) of all articles published in that decade. This trend is likely to continue in the 2020s: nearly a third (32.5%) of all published articles (n = 6,454) appeared in the first 2.5 years of the current decade (Fig 2).

### What disciplines have used the Delphi method the most?

**Fig 3:**
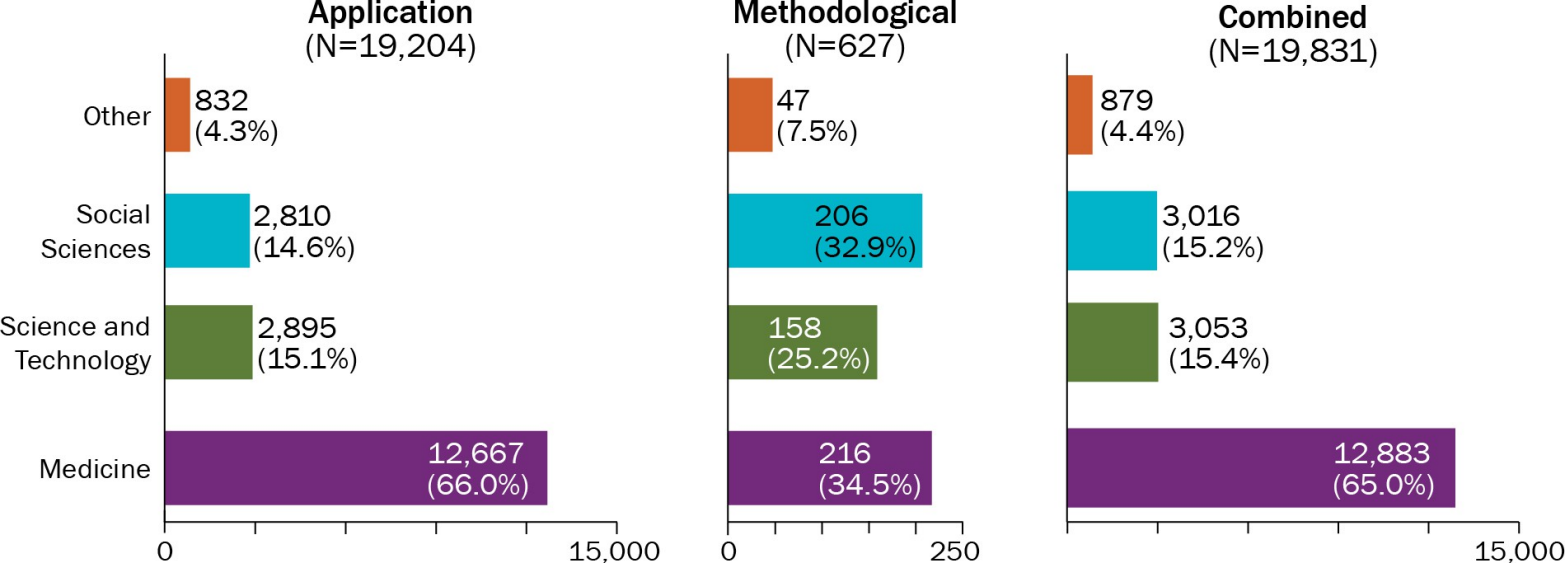
Number of published Delphi articles by discipline. This figure shows the number and percent of Delphi articles published by discipline, including medicine, science and technology, social sciences, and other, separately for Delphi application and methodological articles, as well as combined.

Roughly two-thirds (65.0%, n = 12,883) of all published Delphi articles have appeared in medical journals. By comparison, roughly 15% of all articles appeared in science and technology (n = 3,053) or social science (n = 3,016) journals. The remaining 4.4% (n = 879) appeared in other fields such as arts, music, education, library, and military science (see Fig 3). This finding is identical for application articles, but different for methodological publications. Medicine and social science journals each published roughly a third, science and technology journals published a quarter, and other disciplines published the remaining 4% of articles about the Delphi method.

Moreover, out of ten journals that published the highest number of Delphi articles, six were medical journals (Table 1). Although *BMJ Open* published the largest number of Delphi articles (n = 300), it is followed by *Sustainability* (n = 241), *PLOS ONE* (n = 226), and *Technological Forecasting and Social Change* (n = 199), which are social studies and science and technology journals.

**Table 1:** Top ten journals that published Delphi articles.

Journal Name	Number of Delphi Articles	Discipline
BMJ Open	300	Medicine
Sustainability	241	Social studies
PLOS ONE	226	Science and Technology
Technological Forecasting and Social Change	199	Science and Technology
Trials	120	Medicine
BMC Health Services Research	111	Medicine
International Journal of Environmental Research and Public Health	106	Medicine
Journal of Advanced Nursing	107	Medicine
Annals of Rheumatic Diseases	95	Medicine
Journal of Cleaner Production	90	Science and Technology

Given the large share of medical journals, we looked at their WorldCat subclasses and found the journal subclass information for 92.8% of medical articles (n = 12,883). Journals classified as internal medicine published 28.2% of all Delphi articles that appeared in medical journals and 18.3% of all published Delphi articles (n = 3,626). General medicine journals published 18.0% (n = 2,323) of all Delphi articles in medical journals, followed by “public aspects of medicine” journals that published 12.0% (n = 1,551) of all medical articles that used the Delphi method. Other than these, only surgery journals had more than 1,000 published articles (8.0% of the medicine articles); all other medical journals published no more than 6.0% of the Delphi medical articles.

### How has use of the Delphi method by different disciplines changed over time?

**Fig 4:**
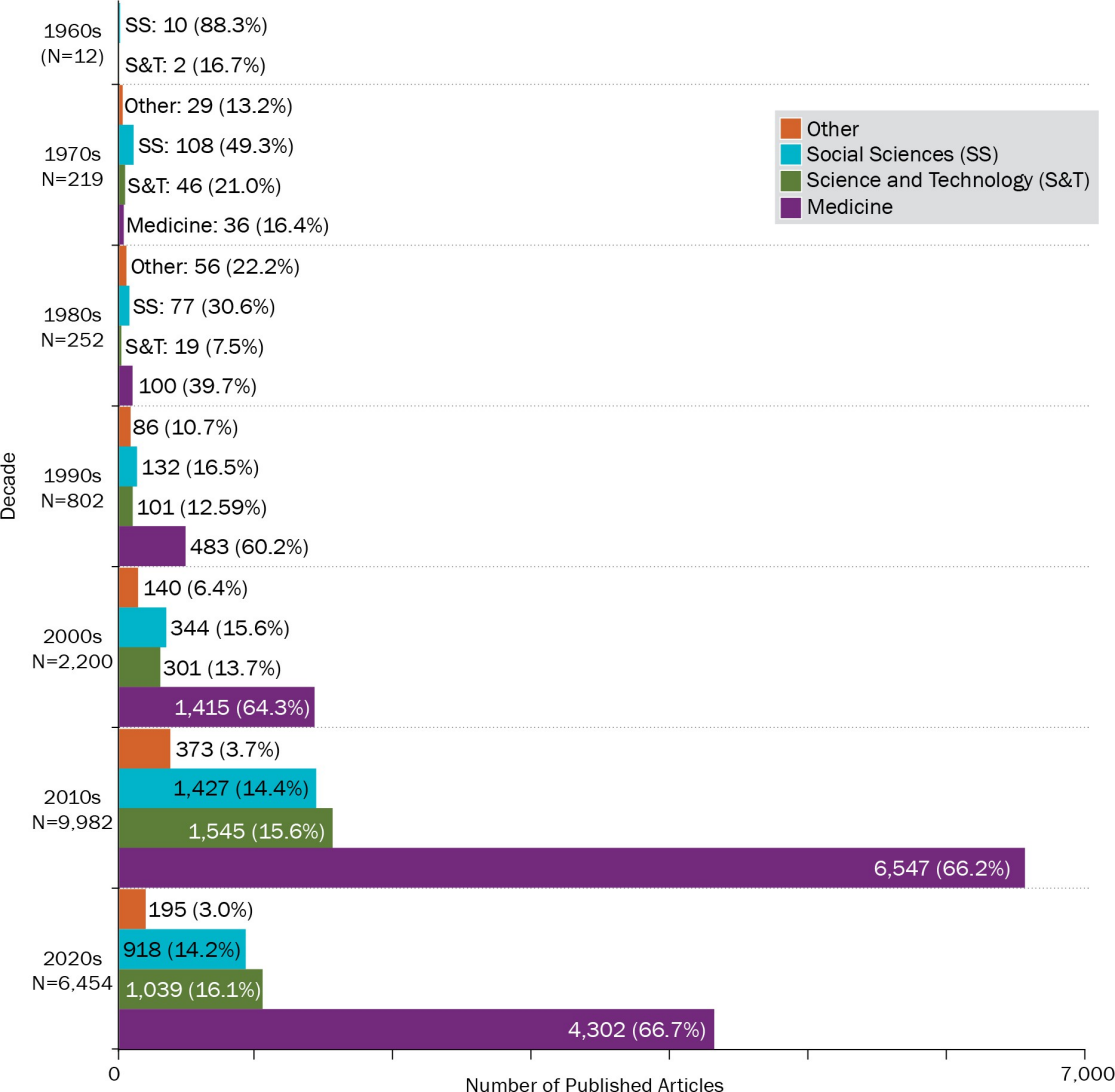
Number of published Delphi articles by decade and discipline. This figure shows the number and percent of Delphi articles published by discipline, including medicine, science and technology, social sciences, and other, in each decade, starting from the 1960s.

The above-described findings show that the expanded use of the Delphi method has been driven by the medical field. While there were no Delphi publications in medical journals in the 1960s and only 16.4% of all Delphi articles published in the 1970s appeared in medical journals, this proportion increased to 39.7% in the 1980s (Fig 4).

Starting from the 1990s, more than 60% of all Delphi articles were published in the medical journal in each decade. Two-thirds of all Delphi articles were published in medical journals in the past 22 years. At the same time, social sciences—which used the Delphi method the most in the 1960s (83.3%) and 1970s (49.3%)—accounted for less than 15% of all Delphi articles published in the past 22 years. The share of Delphi articles published in science and technology journals similarly fluctuated around 15% starting from the 1990s.

In addition to looking at the absolute number of Delphi articles published in medical journals, we also looked at their proportion, relative to the number of all English language medical articles published between 1970 and 2019 and indexed in PubMed. We also compared decennial growth rates in the number of Delphi articles and all PubMed-indexed articles. Table 2 shows that the proportion of Delphi articles was very small (average across all decades is only 0.04%); however, it grew each decade and reached a peak of 0.07% in the 2010s. Nonetheless, the decennial growth rates in the number of published Delphi articles were much higher than those for all published medical articles indexed in PubMed. While the number of Delphi and all published articles grew each decade, the average decennial growth rate for Delphi articles (321%) was 5.5 times higher than that of all PubMed-indexed articles (58%).

**Table 2:** Articles indexed in PubMed, by topic and decade.

	Delphi Articles		All Articles		Proportion of Delphi Articles
Decade	N	Growth Rate	N	Growth Rate	
1970s	22	--	1,648,427	--	0.00%
1980s	93	323%	2,564,843	56%	0.00%
1990s	421	353%	3,846,419	50%	0.01%
2000s	1,343	219%	5,915,383	54%	0.02%
2010s	6,587	390%	10,114,548	71%	0.07%
**Average**	**1,693.2**	**321%**	**4,817,924**	**58%**	**0.04%**

Note: There were no Delphi articles published in the journals indexed in PubMed in the 1960s. We did not include the first 2.5 years of the 2020s to ensure that all time intervals listed include 10 years.

## Discussion

Although the first journal article on the Delphi method was published 60 years ago, an initially slow uptake of the Delphi method accelerated substantially in the past two decades. Namely, it took 34 years to publish the first 1,000 Delphi articles, 7 years to publish the second set of 1,000 articles, and 4 years to publish another 1,000 articles. By comparison, 1,466 Delphi articles were published in the first half of 2022 alone.

Given that the development of the Delphi method was funded by the United States Air Force, early adopters of the Delphi method included military researchers who published their early work that dates back to late 1940s in RAND reports rather than journal articles due to national security concerns [2]. Launches of two journals in late 1960s that focus on the methodology and practice of future-oriented research and technological forecasting—*Futures* and *Technological Forecasting and Social Change*—helped popularize the Delphi method among social scientists and technologists who applied it to a range of complex social problems. These two journals continue to be at the forefront of methodological work on the Delphi method: combined, they published 14.8% of all methodological articles. Concentrated in social science research in the 1970s, the use of the Delphi method shifted toward medical research starting in the 1980s as medical scientists, clinicians, and health services researchers facilitated large-scale diffusion of the Delphi method.

Indeed, medicine is the discipline that has used the Delphi method the most starting from the 1990s. Although the proportion of medical articles indexed in PubMed that use Delphi method remains very low, the decennial growth rate in the number of PubMed-indexed Delphi articles outpaced the growth rates of all medical articles indexed in that database by at least 4 times throughout the past five decades. Medical researchers use Delphi for a wide range of tasks, including the development of standardized sets of outcomes and composite outcome measures used in clinical trials [13], curriculum for medical education research [14], reporting guidelines [15], patient and stakeholder engagement [16], care quality indicators [17]. The development of health care quality measurement as a field is closely connected with the use of the Delphi method and creation of a modified-Delphi method called the “RAND/UCLA Appropriateness Method” (RAM) [18]. The first article that mentioned “appropriateness” in the title or abstract in our study was published in 1986. It focused on describing a new method of expert elicitation to determine appropriateness of different medical and surgical procedures in the absence of evidence from randomized controlled trials. RAND researchers applied Helmer’s epistemological approach to inexact sciences to the medical field [19]. The years of methodological work on the RAM culminated in a publication of the users’ manual for conducting modified-Delphi panels in 2001 [18]. RAM had a profound impact on how health care quality has been assessed and how clinical practice guidelines have been developed around the world [20].

A key contribution of our study is that it updated previous bibliographic analyses of the Delphi method publications that focused on forecasting articles published between 1975 and 2017 [11, 12]. Our analysis identified a much larger number of Delphi articles (19,831 vs. 2,621), partly because we included articles that used the term Delphi not only in the title, but also in the abstract and did not focus only on forecasting articles. As such, our analysis not only replicated, but also expanded on the previous results. Specifically, our findings comprehensively demonstrate that the academic acceptance of the Delphi method is strong across different disciplines, and especially in the medical field; that the interest in the Delphi method grew exponentially in the past 30 years; and that the growth in the number of medical Delphi articles outpaced the growth of all published articles indexed in PubMed. Furthermore, our results show that the articles that applied the Delphi method in empirical research represent an even larger proportion of all publications than previously reported (96.8% in our study vs 90.9% as reported previously [11]). While some of this may be explained by the differences in the search strategies, it also highlights the overall trend towards the growing use of the Delphi method in applied work and a somewhat steady number of published methodological papers.

Our findings also reinforce the trend identified in previous research that shows that recent increases in the use of the Delphi method are being driven largely by the medical and health care fields and that increases in the use of this method are seen broadly across nearly every analyzed discipline. Finally, there has been some reshuffling among the journals publishing the largest number of Delphi articles. Our findings show that *BMJ Open* (a medical journal), rather than *Technological Forecasting and Social Change* (a social science journal) leads in the number of published Delphi articles. In addition, *Sustainability*, a social science journal, and *PLOS ONE*, a science and technology journal, both published a larger number of Delphi articles than *Technological Forecasting and Social Change* (which published the 4^th^ number of Delphi articles). Moreover, the proportion of medical journals among the top 10 journals also increased from 40% to 60% [11]. This finding is not surprising given the widespread use of the method in medical research. Although the medical field has seen a steady growth in the number of PubMed-indexed articles, the rate of decennial growth of the Delphi articles published in medical journals outpaced the overall publication growth in this field, which further reinforces the growing importance of the Delphi method for the medical field.

Given the extensiveness of our search and resource constraints, our study aimed to describe general trends in the use of the Delphi method, rather than to illustrate how different disciplines operationalized or adapted the method over time. It is based on the review of titles and abstracts rather than full text of peer-reviewed journal articles published only in English. Instead of relying on the approach to identifying journal disciplines used in previous studies about the Delphi method [11, 12], we used the LC Subject Classification because it offered an objective approach to classification and had the largest number of the journals in our sample represented in its system. Although doing so complicated direct comparisons with the previous work, we believe that our approach is more robust, objective, and replicable. We note, however, that not all journals were covered by the LC Subject Classification, and we had to manually classify some journals into disciplines. Moreover, we did not find journal subclassifications for 9.2% of articles published in medical journals and excluded them from our subclassification analysis. Our growth trend analysis was limited to the articles indexed in PubMed. Because many articles can also be indexed in other databases, we cannot guarantee that our deduplication process always deleted duplicates from other databases. As such, our growth trend analysis might have undercounted Delphi articles included in the PubMed database. Finally, we did not analyze page counts or the number of citations to the included articles; incorporating these metrics as proxies of value and impact of the Delphi method [11, 12] may be an interesting follow-up analysis.

## Conclusions

Although the expanded use of the Delphi method has been largely driven by the medical field, social scientists and technologists continue to be at the forefront of methodological work on the Delphi method. Therefore, we call for greater transdisciplinary collaboration on the methodological guidance and standards for the Delphi method. Inspired by the existing body of methodological work in the field of systematic reviews and randomized trials, we specifically call for the development of methodological guidance for conducting rigorous Delphi panels, tools for critical appraisal, and standards for reporting studies using the Delphi method that are applicable to a wide range of disciplines rather than a particular sub-filed, such as palliative care [21].

## Supporting information

S1 Data (XLSX)

S1 Appendix Search strategy. (DOCX)
