# Supplementary material for: Disciplinary trends in the use of the Delphi method: A bibliometric analysis
Source: PLoS One. 2023 Aug 15;18(8):e0289009. doi: 10.1371/journal.pone.0289009 (PMC10427003; doi:10.1371/journal.pone.0289009)
Supplement: S1 Appendix — (DOCX) [file pone.0289009.s002.docx]

# Appendix

**Search strategy**

We searched the bibliographic literature covering the years of 1950 through 2022 (May 30/June 1) for the term *delphi* in all citation fields (except where noted below for practical reasons after a determination of irrelevance and irregularities in meta data). We removed duplicate citations, proceedings papers, and citations retrieved by the location of Delphi (Greece) or the use of Delphi detector for physics.

**PubMed
English, 1950-June 1, 2022
search executed 21 June 2022**

Delphi[all fields]

*Results: 15054 – duplicates/location/ = 14979*

**CINAHL Plus via Ebsco (Cumulative Index of Nursing and Allied Health Literature)
phrase searching, English January 1950-May 2022
search executed 21 June 2022**

Delphi

*Results: 8991 – duplicates = 1873*

**APA PsycInfo via Ebsco
phrase searching, English January 1950-May 2022
search executed 21 June 2022**

"Delphi"

*Results: 2734 – duplicates= 776*

**AMED via Ebsco (Allied and Complementary Medicine Database)
phrase searching, English January 1950-May 2022
search executed 21 June 2022**

Delphi

*Results: 404 – duplicates= 21*

**ACM - The ACM Guide to Computing Literature
English, 1950-May 2022, Research Articles
Search executed 22 June 2022**

[Title: delphi] OR [Abstract: delphi]

*Results: 236 – remove proceedings papers/duplicates = 34*

**Scopus (mulitidisciplinary)**
**English, 19~~6~~50 – 1980, Article, Review**
**Search executed 22 June 2022**

Delphi

*Results: 651 – duplicates = 583*

**ERIC via Ebsco (Education Resource Information Center)
English; January 1950-May 2022; phrase searching; Academic Journals/ERIC documents/Reports
Search executed 22 June 2022**delphi

*Results: 1850 - duplicates = 1102*

**Business Source Complete via Ebsco
English; January 1950-May 2022; phrase searching; Academic Journals
Search executed 22 June 2022**

delphi

*Results: 2294 - duplicates = 1491*

**Web of Science (multidisciplinary)**
**Searched: Science Citation Index Expanded (SCI-EXPANDED), Social Sciences Citation Index (SSCI), Arts & Humanities Citation Index (A&HCI), Emerging Sources Citation Index (ESCI) | Limited to: articles (only), review articles, early access; English Language | 1980-June 1, 2022.**
**26 June 2022**

TS=delphi
NOT
(AB=delphi detector) OR (GP=delphi)

*Results: 18720 -duplicates/location/non-relevant = 5372*

**ROCS (RAND Library Catalog)
Search executed 21 June 2022**

(Word (delphi) AND (Collection Type is RAND Publications) AND (Word (dis=3) NOT (Media Type is EXTPUB)

(Word (delphi) AND (Collection Type is RAND Publications) AND (Word (dis=1) NOT (Media Type is EXTPUB)

(Word (delphi) AND (Collection Type is RAND Publications) AND (Word (dis=2) NOT (Media Type is EXTPUB)

*Results: 197 (public); 5 (FOUO); 54 (internal) = 256*
